# Jackdaws Use Lynx Scat in Nests: Implications for Iberian Lynx Genetic Monitoring

**DOI:** 10.1002/ece3.71859

**Published:** 2025-07-24

**Authors:** José Jiménez, Rafael Finat, Mario Fernández‐Tizón, Javier Hernández‐Hernández, Alicia I. Martínez‐González, Emilio Virgós

**Affiliations:** ^1^ Instituto de Investigación en Recursos Cinegéticos (IREC, CSIC‐UCLM‐JCCM) Ciudad Real Spain; ^2^ Finca el Castañar Mazarambroz Toledo Spain; ^3^ Área de Biodiversidad y Conservación, Departamento de Biología, Geología, Física y Química Inorgánica Rey Juan Carlos University Madrid Spain; ^4^ Road Ecology Lab, Department of Biodiversity, Ecology and Evolution, Faculty of Biology Complutense University of Madrid Madrid Spain

**Keywords:** conservation, genetic monitoring, interspecific interactions, jackdaws, lynx scat, spatial capture‐recapture

## Abstract

We present the first documented case of jackdaws ( *Coloeus monedula* ) collecting and placing Iberian lynx ( *Lynx pardinus* ) scat in their nests in the Montes de Toledo, Spain. This behavior may significantly compromise conservation efforts for species whose monitoring relies on non‐invasive genetic sampling—such as the lynx—especially in areas with dense populations of jackdaws or other species exhibiting similar behavior, where this removal may substantially reduce sample availability. Using artificial nest boxes equipped with camera traps, we confirmed that jackdaws actively transport lynx scat to their nests. In a controlled experiment simulating a lynx latrine, all scat was removed in just over an hour. Simulations using spatial capture‐recapture (SCR) models showed that this behavior can introduce bias and reduce the accuracy of population estimates based on genetic sampling, a widely used method in wildlife monitoring. These findings highlight the importance of considering interspecific interactions when designing monitoring protocols for threatened species. More broadly, this case illustrates how overlooked ecological behaviors can compromise conservation tools and underscores the need for adaptive monitoring strategies in dynamic ecosystems.

## Graphical abstract

This study documents the novel behavior of jackdaws ( *Coloeus monedula* ) collecting Iberian lynx ( *Lynx pardinus* ) scat for nest construction in central Spain. This behavior significantly impacts non‐invasive genetic monitoring by reducing scat availability, thereby introducing bias and reducing the accuracy of spatial capture‐recapture (SCR) population estimates. The findings highlight the need to account for interspecific interactions in conservation monitoring protocols.

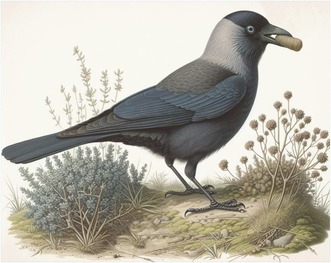

## 1 Introduction

In 2023, while contributing to the report ‘Metodología para el seguimiento genético no invasivo del lince ibérico’ (Godoy et al. 2024) for the LIFE Lynxconnect programme, one of its authors (J. Jiménez) was alerted by R. Finat, co‐author of this note, to jackdaws actively collecting lynx scat. This behaviour was documented via camera traps in six different photographic series (see Appendix S1). Although initially anecdotal, this observation soon revealed potential implications for conservation monitoring.

**FIGURE 1:**
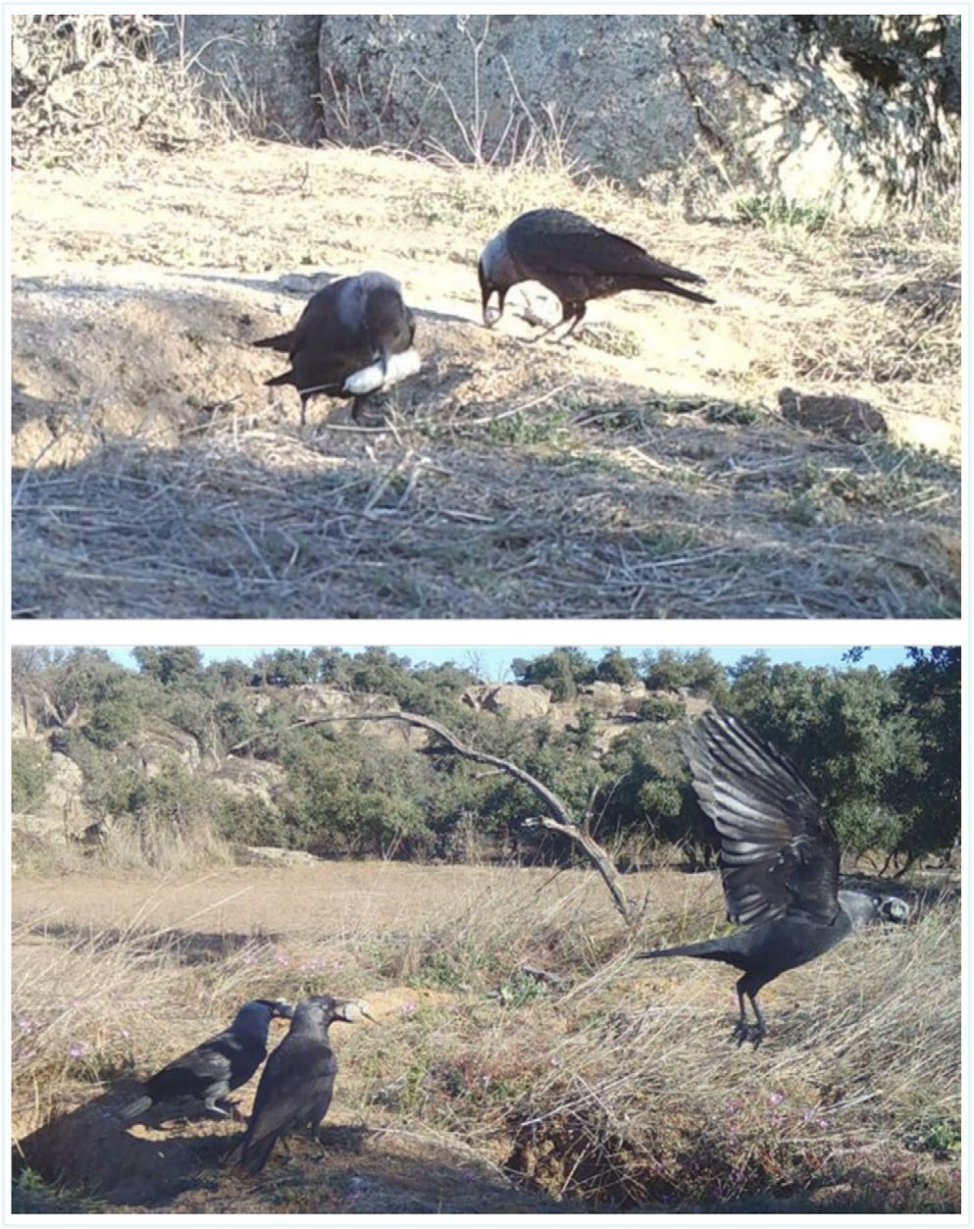
Western jackdaws ( *Coloeus monedula* ) collecting and transporting Iberian lynx ( *Lynx pardinus* ) scat at a lynx latrine near rabbit burrows in the Toledo Mountains, Spain. Top: 8 March 2023, 09:59 AM; Bottom: 7 April 2023, 09:59 AM.

These photographs show several jackdaws at a lynx latrine, near rabbit ( *Oryctolagus cuniculus* ) burrows. Each bird is seen carrying lynx scat in its beak. In other images, the birds are observed in flight, transporting the scat (see Figure 1 and Appendix S1). The behavior appeared systematic and intentional, rather than incidental or exploratory. Similar behavior was also observed in magpies ( *Pica pica* ) within the study area.

The jackdaw is a medium‐sized corvid known for its advanced cognitive abilities, problem‐solving skills, and documented tool use (Jacobs and Osvath 2023). Measuring 34–39 cm in length, it has predominantly black plumage with a contrasting grey nape and pale‐grey irises. Highly social and vocal, jackdaws form structured groups with complex social hierarchies. Their adaptability allows them to thrive in a wide range of environments, including agricultural areas, open woodlands, coastal cliffs, and urban settings.

The Iberian lynx is an iconic species in Spanish conservation. Once on the brink of extinction in the 1980s, with an estimated 30 breeding females (Guzmán et al. 2004), its population has increased to an estimated 1299 individuals in 2023 thanks to intensive management efforts, including captive breeding and reintroduction (Life Lynxconnect Team 2024). Given the current population size, individual identification through camera traps has become logistically unfeasible. Therefore, monitoring based on non‐invasive genetic sampling and spatial capture‐recapture (SCR) methods is being considered (Godoy et al. 2024).

Our initial hypothesis was that jackdaws were transporting lynx scat into their nests, but we needed to document the transport of lynx scat to the nests. To our knowledge, the use of carnivore scat in bird nests has only rarely been documented. One example is the common waxbill ( *Estrilda astrild* ) in Africa, which gathers carnivore scat—often containing indigestible hair and bones—and places it in, on, and around its nest (Schuetz 2005). Goodwin (1982) suggested this behavior may serve to camouflage nests or deter predators, while Schuetz (2005) found that nest material selection could influence nesting success, likely by reducing predation risk. Another case involves the firewood‐gatherer ( *Anumbius annumbi* ) in the Pampas of South America, where scat use has been interpreted as a decorative behavior to attract mates (Delhey et al. 2017). Sheard et al. (2023) also reported the use of droppings as a binder in nests, linking this behavior to beak morphology, with binder materials typically used by species with especially bulky bills.

It is well established that some bird species incorporate a wide variety of materials—such as flowers, lichens, and artificial objects—into their nests. Proposed explanations include sexual selection (as an extended phenotype of male quality), parasite avoidance, insulation, protection, and, less commonly, predator deterrence (Amo et al. 2008; Clark 1991; Fauth et al. 1991). We aimed to investigate the extent of this behavior in jackdaws—potentially amplified by the social dynamics of this gregarious species, and assess how their collection of scat could affect lynx population estimates based on non‐invasive genetic sampling and SCR methods. Given the novelty of this behavior and the limited empirical evidence available, this study should be considered exploratory in nature. Our aim is to document and illustrate a potentially overlooked ecological interaction, rather than to provide definitive estimates of its prevalence or impact.

## 2 Methods

In 2023, field observations were conducted in Los Montes de Toledo, central Spain, where the collection of Iberian lynx scat by jackdaws was first observed. Four jackdaw nests located in cavities of evergreen oaks ( *Quercus ilex* ) were inspected. A scat fragment—possibly from a lynx—was found in one nest, although its origin could not be confirmed due to its degraded condition.

Studying material provisioning in natural cavities is challenging due to their inaccessibility. For this reason, we decided to use nest boxes. In 2024, we installed 29 wooden nest boxes (60 × 30 × 30 cm; entrance diameter: 7 cm) at heights of 2.5–4 m in mature evergreen oaks within the area where the behavior had been observed. Of these, 24 were fitted with camera traps to monitor nest‐building activity. The nest boxes were inspected after the breeding season.

To assess the rate and timing of scat collection, an artificial lynx latrine was created by relocating scat from natural latrines to a site within 500 m of a jackdaw colony, in an area lacking a natural latrine. The simulated latrine was placed in a location typical of lynx behavior—next to a rabbit burrow—and stocked with 19 pieces of lynx scat. We used a camera trap to document interactions and analyse jackdaw behavior. In this study, we did not quantify the global proportion of lynx scats removed by jackdaws. However, to illustrate the potential implications of such removal, we conducted simulations using a customised R script (R Core Team 2025) and the NIMBLE package (de Valpine et al. 2017). These simulations aimed to evaluate the potential impact of scat removal on spatial capture‐recapture (SCR) density estimates. The code used for these simulations is available at: https://zenodo.org/records/15771760. The study area was modelled as an 8 × 8 detector grid with 800 m spacing. We simulated a population of 10 lynx, which is comparable to the estimated number of individuals occupying the study area, thereby ensuring ecological realism while maintaining computational tractability, using a SCR movement parameter (σ) of 1000 m and a baseline detection rate ($\lambda_{0}$) of 0.1, across five sampling occasions.

Sampling was assumed to occur on a cell‐by‐cell basis, with transect samples accumulated at the 64 grid centroids (Jiménez et al. 2024), and equal sampling effort across all cells. A Poisson observation model was applied (see Milleret et al. 2018). Three jackdaw colonies were randomly placed within the grid, and it was assumed that lynx scats within a 2 km radius of each colony (Chambon et al. 2024) were partially removed (25 and 50% scenarios). This assumption is consistent with the land use patterns we have observed in the study area, although we acknowledge that it simplifies spatial heterogeneity by assuming homogeneous use within the 2 km radius. With the same configuration, we simulated a scenario without scat removal (‘control’). One hundred random configurations of lynx activity centres were simulated within the state space, applying a 2.5 σ buffer (2500 m) around the detector grid. We used Bayesian MCMC (Markov Chain Monte Carlo) methods to fit the SCR simulations using the R (R Core Team 2025) package NIMBLE (de Valpine et al. 2017). We specified vague or weakly informative priors for all parameters, and ran three chains for 5000 iterations, with a burn‐in of 1000, resulting in 15,000 posterior samples per parameter. Bias and root mean square error (RMSE) were calculated using the R package SimDesign (Chalmers and Adkins 2020).

## 3 Results

**FIGURE 2:**
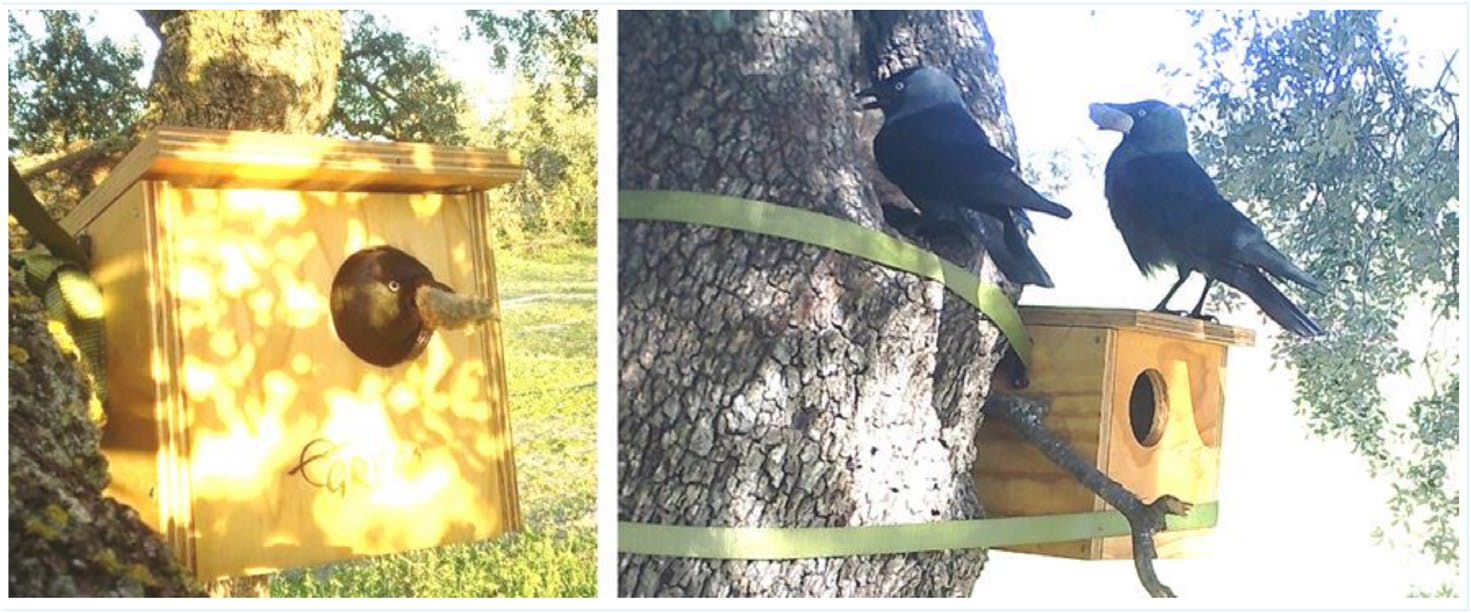
Jackdaws transporting presumed Iberian lynx scat to artificial nest boxes in the Montes de Toledo. Left: 9 May 2024; Right: 17 March 2024. Images captured by camera traps installed to monitor nest material provisioning.

Of the 29 nest boxes installed, five were used by jackdaws during the study period, three of which supported full breeding activity, including egg‐laying, incubation, and chick‐rearing during 2024. In two of these active nests, jackdaws were observed placing scat inside, presumed to be from an Iberian lynx based on size and morphology, as illustrated in Figure 2. The scats found later at the nest were semi‐decomposed but still retained a recognizable structure. Subsequent macroscopic analysis of the nest contents, conducted using a binocular microscope, revealed the presence of hair from both red fox ( *Vulpes vulpes* ) and Iberian lynx. These observations support the hypothesis that jackdaws actively select and incorporate carnivore scat as nesting material. However, while the presence of lynx hairs strongly suggests the inclusion of lynx scat, the detection of fox hairs could originate either from fox scat or from a fox killed by a lynx (Jiménez et al. 2019).

**FIGURE 3:**
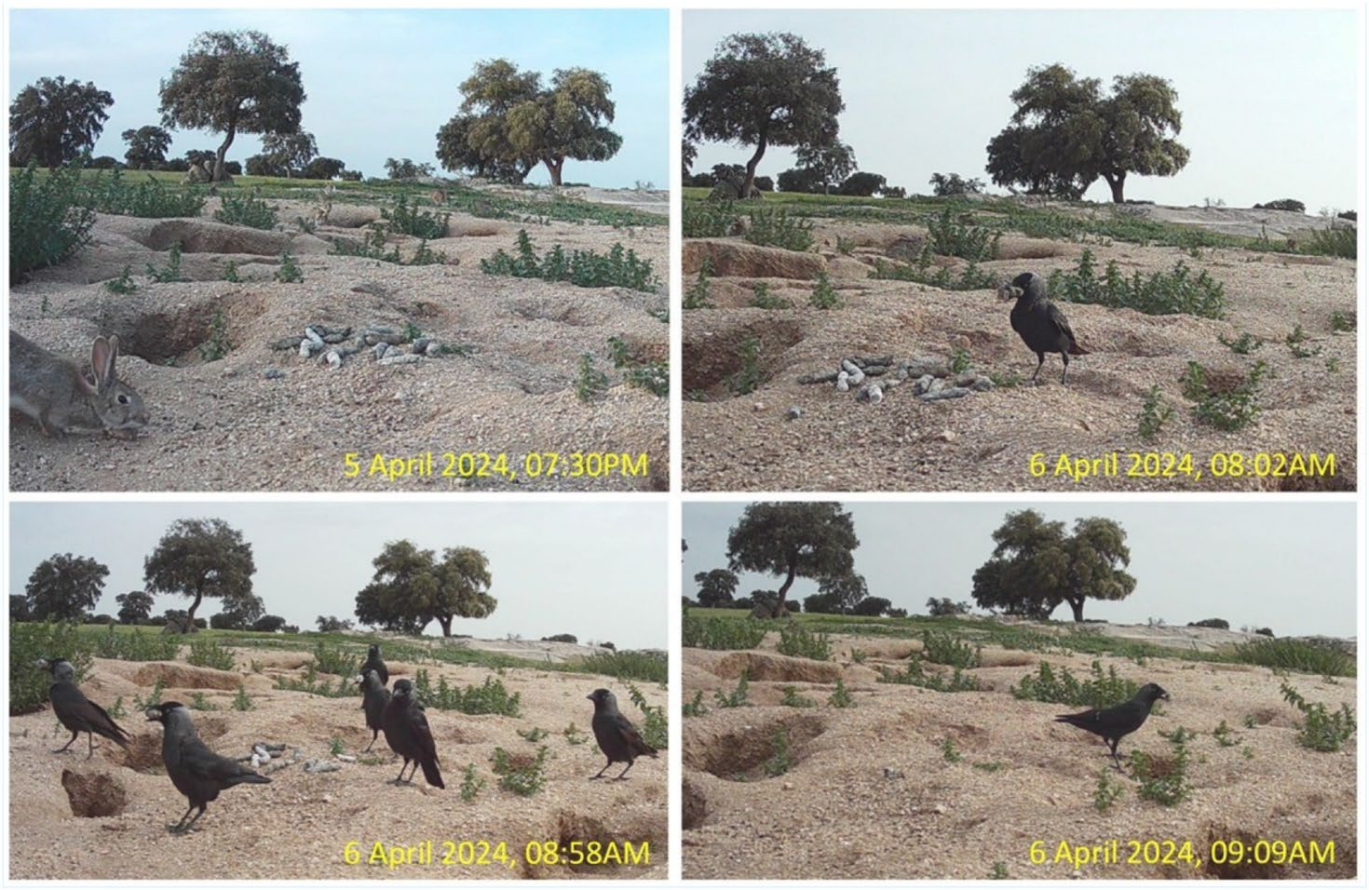
Timeline of lynx scat removal by jackdaws at the artificial latrine site on 6 April 2024. All 19 scat fragments were removed within 67 min of the first jackdaw's arrival, indicating rapid and coordinated collection behavior.

The artificial latrine was prepared by 6:00 PM on 5 April 2024, with scat material placed at the site. A camera trap, triggered by rabbit activity at 7:16 PM, confirmed the presence of the scat (Figure 3). The following morning, on 6 April 2024, at 8:02 AM, the first jackdaw was recorded at the latrine. By 8:58 AM, six jackdaws were observed, three of which were carrying scat in their beaks. At 9:09 AM—just 1 h and 7 min after the first jackdaw appeared—the final scat was removed, as documented by the camera trap (Figure 3 and Appendix S1), mirroring behavior previously observed at natural latrines during lynx camera trapping in 2023.

Simulations assessing the impact of jackdaw‐mediated scat removal on SCR estimates revealed a measurable effect on model performance. In the absence of jackdaw interference (control scenario), the mean bias in lynx population estimates was 0.63, with a RMSE of 4.00. In contrast, scenarios incorporating jackdaw removal of 25% and 50% of scats resulted in mean biases of 1.18 and 1.70, and RMSEs of 5.48 and 11.63, respectively. In all cases, only one simulated dataset was not identifiable, allowing for direct and consistent comparisons across scenarios.

A mean bias of 1.7 indicates that, on average, population estimates deviate by approximately 17% from the true population size. Given the small simulated population (10 individuals), the observed increase in RMSE from 4.00 to 11.63 represents a substantial loss of precision—equivalent to more than 116% of the true population size. Moreover, the increase in bias and RMSE under jackdaw interference highlights the potential for this behavior to destabilize SCR model performance.

## 4 Discussion

The incorporation of carnivore scat into jackdaw nests represents a novel and ecologically meaningful behavior that has not been previously documented. Although the specific ecological drivers behind this behavior remain uncertain, nest construction and material selection are traits shaped by both natural and sexual selection, with implications for reproductive success, predator deterrence, and social signalling. One possible hypothesis is that by incorporating lynx scat, jackdaws may be modifying the microenvironment of their nests in ways that influence incubation conditions or deter potential threats. While this remains speculative, it is consistent with the concept of the nest as an extended phenotype and a tool for niche construction, as discussed by Deeming (2023), where material choice can significantly affect the functional properties of the nest.

This behavior introduces a novel and previously overlooked source of bias in non‐invasive genetic monitoring, particularly in systems that rely on scat‐based data for population estimation. The removal and relocation of scats by jackdaws may alter the spatial distribution and availability of genetic material, thereby compromising the accuracy of demographic models. Although this behavior has so far only been observed in jackdaws and magpies, it is plausible that other colonial corvids—such as the azure‐winged magpie ( *Cyanopica cyanus* ), which shares much of the Iberian lynx's range—may exhibit similar behaviors. If so, the cumulative impact on scat‐based monitoring could be substantial, particularly in areas with high corvid densities.

In the context of endangered species management, the levels of uncertainty introduced by jackdaw interference can critically impair conservation planning. Although there is no universally accepted threshold, a mean bias exceeding 5%–10% is generally considered problematic in conservation contexts if unrecognized (Dettloff 2023). Similarly, an RMSE greater than 30%–40% of the true population size can undermine the reliability of estimates. These values are not formal cut‐offs, but they align with practical expectations for model performance in small or endangered populations (Buckland et al. 2023).

These findings underscore the importance of integrating interspecific behavioral interactions into conservation frameworks. As conservation biology increasingly relies on indirect methods, understanding how species influence the availability and distribution of monitoring data becomes essential. This is particularly true for endangered species such as the Iberian lynx, where accurate population estimates are critical for guiding management and recovery efforts. The heterogeneity introduced by scat removal may be difficult to model explicitly; however, if the number of remaining samples is sufficient for SCR estimation, it could potentially be accounted for by incorporating a random effect at the site level. However, to avoid this potential source of bias in our study area, we recommend conducting SCR sampling during the autumn and winter months, prior to the seasonal arrival of jackdaws. Future protocols should therefore be designed with these ecological dynamics in mind to ensure the robustness and reliability of conservation inferences.

## Author Contributions

**José Jiménez:** conceptualization (equal), formal analysis (lead), funding acquisition (lead), writing – original draft (lead). **Rafael Finat:** conceptualization (lead), data curation (equal), investigation (equal). **Mario Fernández‐Tizón:** conceptualization (equal), data curation (lead), investigation (lead). **Javier Hernández‐Hernández:** conceptualization (equal), data curation (equal), investigation (equal). **Alicia I. Martínez‐González:** data curation (equal), investigation (equal). **Emilio Virgós:** conceptualization (equal), data curation (equal), investigation (equal), writing – original draft (equal).

## Conflicts of Interest

The authors declare no conflicts of interest.

## Supporting information


**Appendix S1:** ece371859‐sup‐0001‐Appendix.pdf.

## Data Availability

All data supporting the findings reported in this study are included in the manuscript. The simulation code is publicly available at https://zenodo.org/records/15771760.
