# Supplementary material for: Jackdaws Use Lynx Scat in Nests: Implications for Iberian Lynx Genetic Monitoring
Source: Ecol Evol. 2025 Jul 24;15(7):e71859. doi: 10.1002/ece3.71859 (PMC12289391; doi:10.1002/ece3.71859)
Supplement: Supplementary file 1 — Appendix S1: ece371859‐sup‐0001‐Appendix.pdf. [file ECE3-15-e71859-s001.pdf]

Appendix

Selected camera trap images of jackdaws collecting Iberian lynx scat, recorded in the Toledo Mountains (Toledo, Spain; 2019–2024)

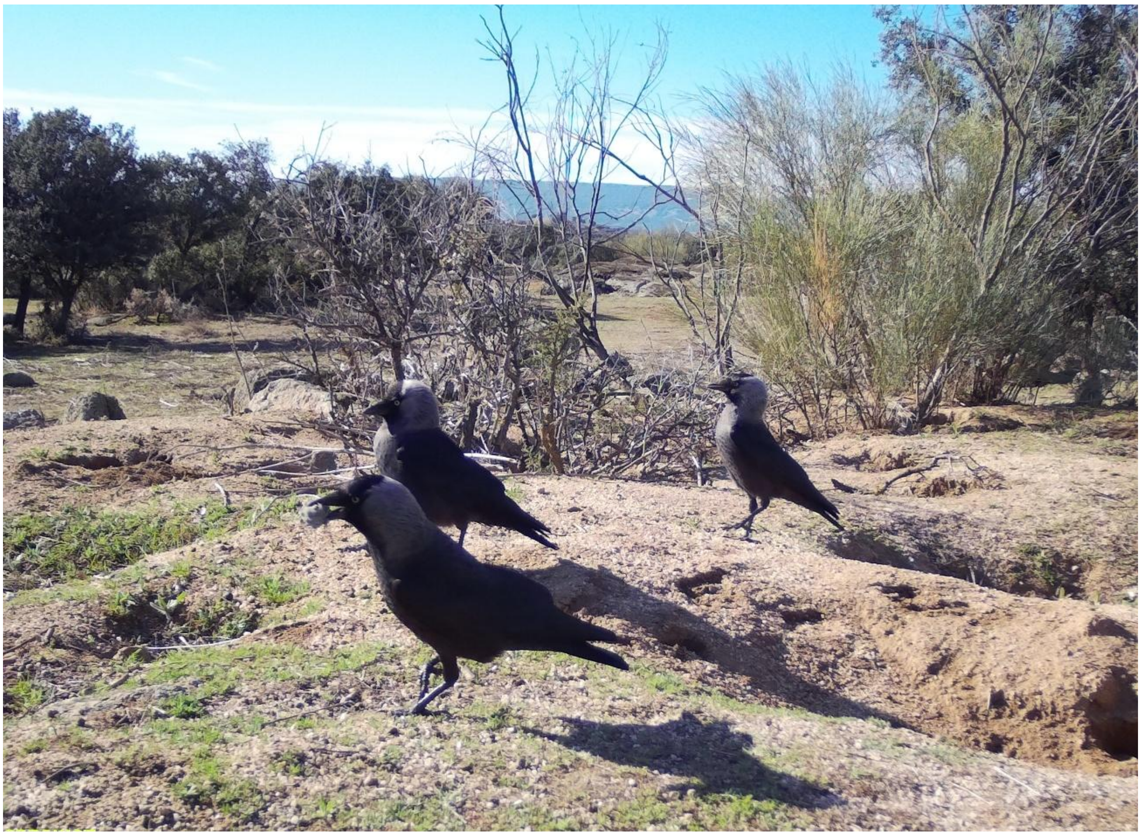

30/04/2019

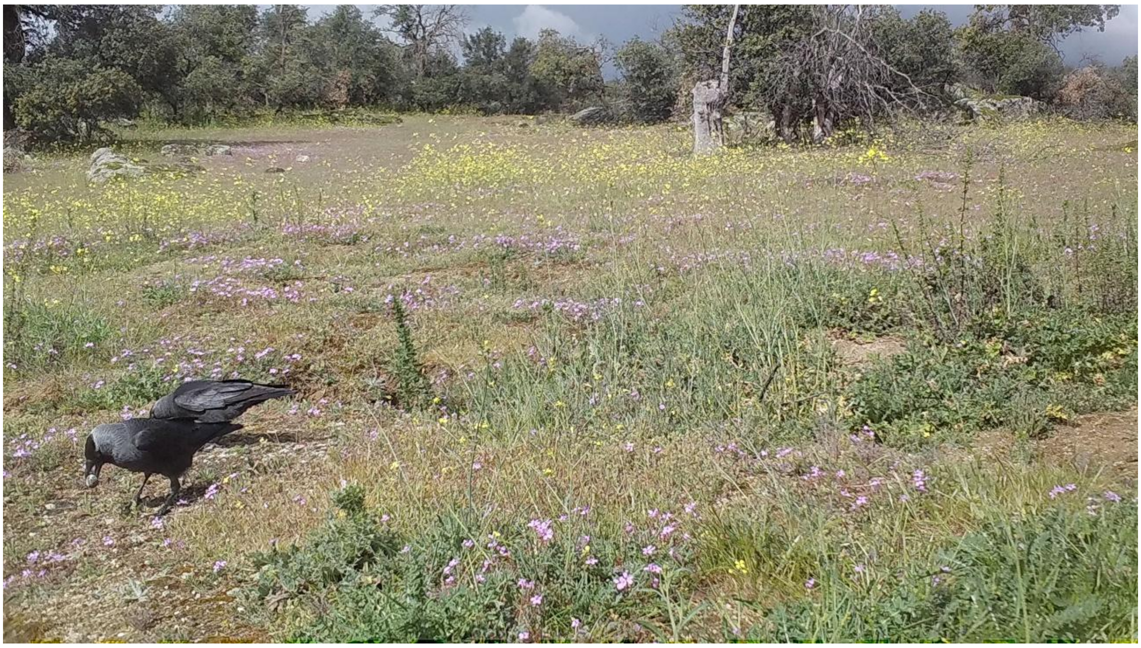

11/04/2021

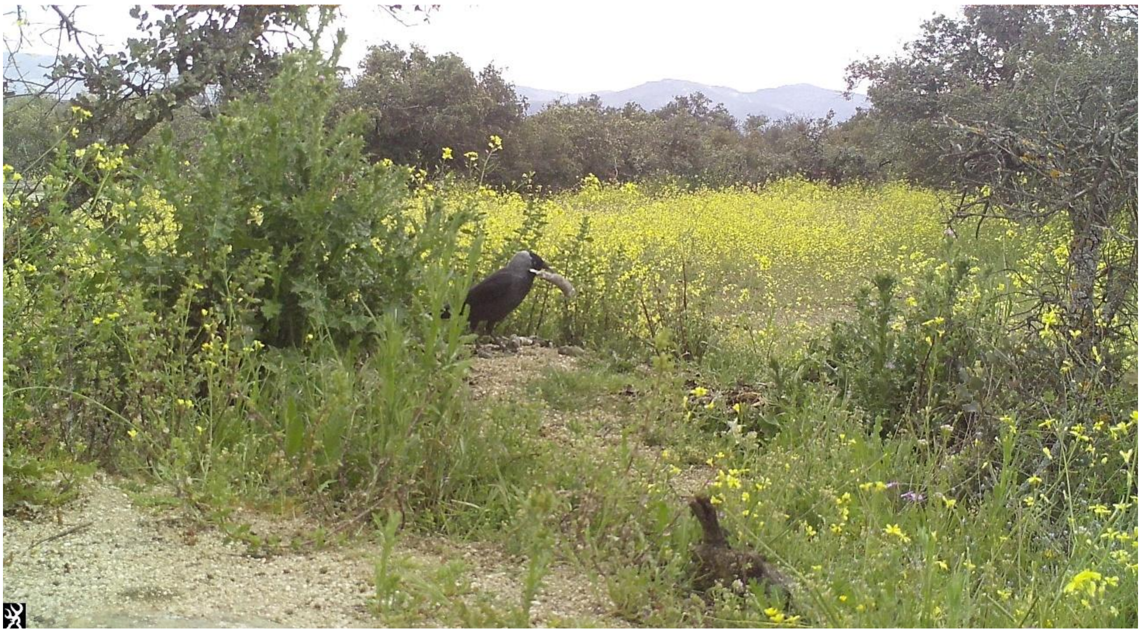

26/04/2022

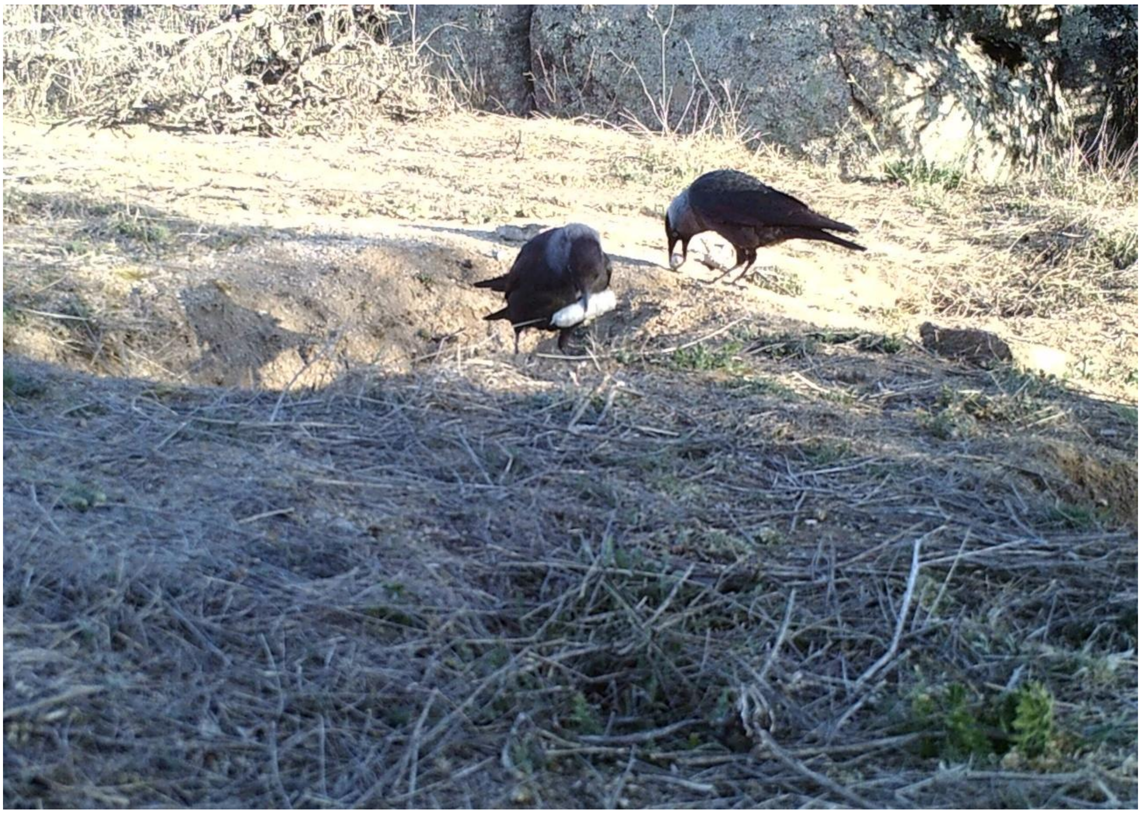

08/03/2023

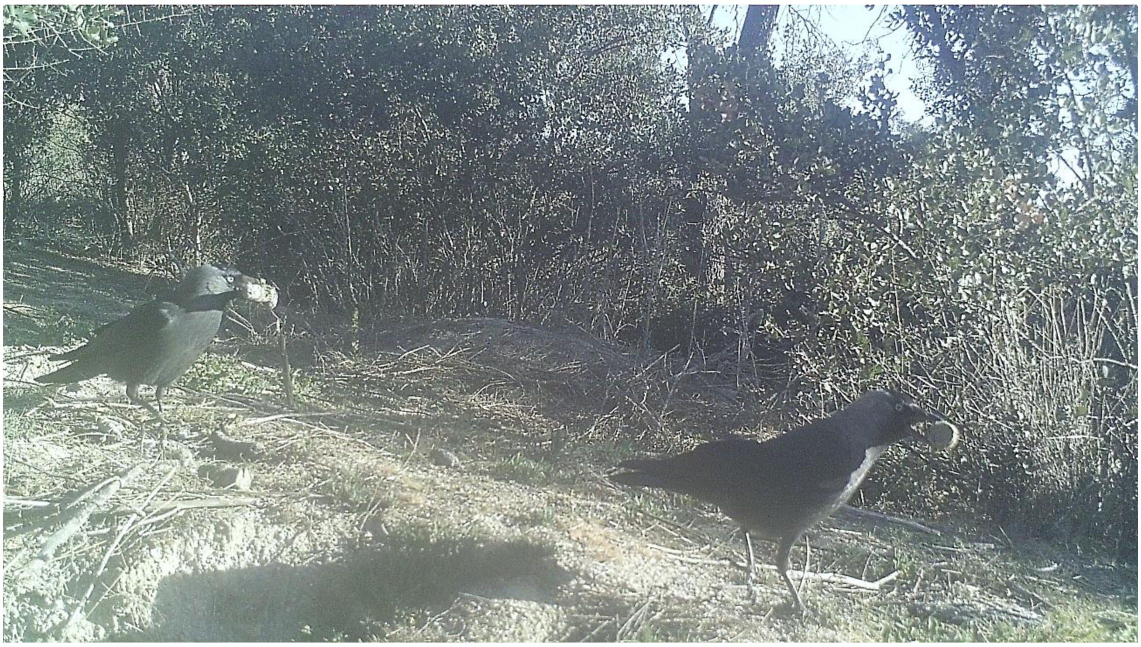

07/04/2023

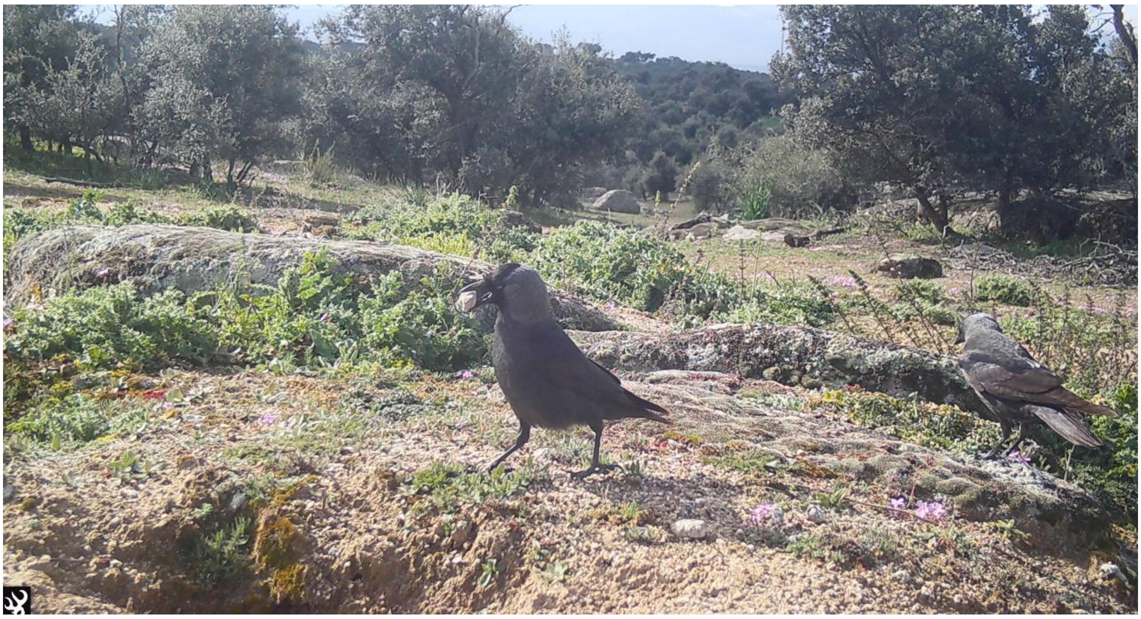

14/03/2024
